# Increased risk of ischemic heart disease, hypertension, and type 2 diabetes in women with previous gestational diabetes mellitus, a target group in general practice for preventive interventions: A population-based cohort study

**DOI:** 10.1371/journal.pmed.1002488

**Published:** 2018-01-16

**Authors:** Barbara Daly, Konstantinos A. Toulis, Neil Thomas, Krishna Gokhale, James Martin, Jonathan Webber, Deepi Keerthy, Kate Jolly, Ponnusamy Saravanan, Krishnarajah Nirantharakumar

**Affiliations:** 1 School of Nursing, Faculty of Medical and Health Sciences, University of Auckland, Auckland, New Zealand; 2 Institute of Applied Health Research, University of Birmingham, Birmingham, United Kingdom; 3 Department of Diabetes, University Hospitals Birmingham NHS Foundation Trust, Birmingham, United Kingdom; 4 Diabetes, Endocrinology & Metabolism, Division of Health Sciences, Warwick Medical School, University of Warwick, Coventry, United Kingdom; University of Cambridge, UNITED KINGDOM

## Abstract

**Background:**

Gestational diabetes mellitus (GDM) is associated with developing type 2 diabetes, but very few studies have examined its effect on developing cardiovascular disease.

**Methods and findings:**

We conducted a retrospective cohort study utilizing a large primary care database in the United Kingdom. From 1 February 1990 to 15 May 2016, 9,118 women diagnosed with GDM were identified and randomly matched with 37,281 control women by age and timing of pregnancy (up to 3 months). Adjusted incidence rate ratios (IRRs) with 95% confidence intervals (CIs) were calculated for cardiovascular risk factors and cardiovascular disease. Women with GDM were more likely to develop type 2 diabetes (IRR = 21.96; 95% CI 18.31–26.34) and hypertension (IRR = 1.85; 95% CI 1.59–2.16) after adjusting for age, Townsend (deprivation) quintile, body mass index, and smoking. For ischemic heart disease (IHD), the IRR was 2.78 (95% CI 1.37–5.66), and for cerebrovascular disease 0.95 (95% CI 0.51–1.77; *p-*value = 0.87), after adjusting for the above covariates and lipid-lowering medication and hypertension at baseline. Follow-up screening for type 2 diabetes and cardiovascular risk factors was poor. Limitations include potential selective documentation of severe GDM for women in primary care, higher surveillance for outcomes in women diagnosed with GDM than control women, and a short median follow-up postpartum period, with a small number of outcomes for IHD and cerebrovascular disease.

**Conclusions:**

Women diagnosed with GDM were at very high risk of developing type 2 diabetes and had a significantly increased incidence of hypertension and IHD. Identifying this group of women in general practice and targeting cardiovascular risk factors could improve long-term outcomes.

## Abstract 2

Krishnarajah Nirantharakumar and colleagues present results from a cohort of women with gestational diabetes and identify these as a target group for preventative interventions for cardiovascular disease and type 2 diabetes.

## Author summary

**Why was this study done?:**

- The prevalence of gestational diabetes mellitus (GDM) is increasing rapidly in most developed countries.
- Although it is well documented that women diagnosed with GDM have a greatly increased lifetime risk of developing type 2 diabetes, there is a paucity of reports on its association with cardiovascular disease.
- Only 3 previous population-based cohort studies have reported on GDM and the long-term risk of cardiovascular events (including 1 utilizing a primary care database), and no studies have been reported from the United Kingdom (UK).

**What did the researchers do and find?:**

- This population-based retrospective cohort study utilized a large UK primary care database that included more than 9,000 women diagnosed with GDM between 1 February 1990 and 15 May 2016.
- The association between GDM and the development of type 2 diabetes, hypertension, and cardiovascular disease postpartum was examined.
- Women diagnosed with GDM were over 20 times more likely to develop type 2 diabetes, 2.8 times more likely to develop ischemic heart disease, and twice as likely to develop hypertension, although no association was found for cerebrovascular disease.
- Less than 60% of women diagnosed with GDM were screened in primary care for type 2 diabetes in the first year postpartum, and the proportion screened rapidly declined after this period.
- Screening rates were also low for cardiovascular risk factors such as smoking, high body mass index, hypertension, and dyslipidemia, and were similar to the screening rates in control women.

**What do these findings mean?:**

- GDM in women increases the risk for hypertension and ischemic heart disease in addition to type 2 diabetes.
- Although the National Institute for Health and Care Excellence guidelines recommend annual screening for type 2 diabetes in women diagnosed with GDM, this study found that follow-up screening was poor for type 2 diabetes and other cardiovascular risk factors such as hypertension.
- Women diagnosed with GDM are an identifiable group of at-risk women and ideal for targeting preventative metabolic and cardiovascular interventions.
- Clinical guidelines should include postpartum screening and management for all cardiovascular risk factors in women diagnosed with GDM and not restrict it to diabetes.

## Introduction

Gestational diabetes mellitus (GDM) is increasing, largely due to the obesity epidemic [1] and increasing maternal age [2]. Although inconsistencies exist across countries for screening for GDM [1] and diagnostic cutoff points for the oral glucose tolerance test [3], reported prevalences are 2%–6% for Europe [1], 7% for North America [4], and 1%–9% and 4%–24% for white British and South Asian (SA) women, respectively, in England and Southern Ireland [5], reflecting the higher 10%–20% prevalence in high-risk populations [2]. It is well accepted that the early identification and treatment of women with GDM reduces pregnancy and perinatal complications [1,6,7] and improves infant birth weights [8]. Women with GDM are also more likely to have markers for insulin resistance and beta cell dysfunction [9–13], particularly if overweight [14], and GDM is a well-established predictor for progression to type 2 diabetes and carries up to a 70% lifetime risk [15].

Although the association between GDM and type 2 diabetes is well established [15,16], onset of the latter following delivery is less well documented and understood in terms of underlying genetic and lifestyle factors [16]. Only 3 previous large population-based studies quantifying the increased risk of cardiovascular disease following delivery in women diagnosed with GDM were identified [17–19]. One Canadian retrospective study identified 8,191 women with GDM and age-matched them with 81,262 control women without GDM utilizing primary care records in Ontario [17]. One French study utilizing hospital records identified over 1.5 million women who delivered infants during 2007 and 2008 and included 62,958 women with GDM who were compared with all women without GDM who delivered a healthy infant during that period [18]. More recently, the North American Nurses’ Health Study II group reported on 5,992 nurses who self-reported a history of GDM from a total cohort of 89,479 [19].

All 3 studies reported an increase risk of cardiovascular events for women with GDM compared with women without GDM during the 12, 7, and 26 years of postpartum follow-up for the 3 studies, respectively. The increased risk for major cardiovascular events in people with type 2 diabetes in addition to other traditional risk factors [15,20] and at an early age [21] is well documented. Despite this, and the recommendation for annual screening for type 2 diabetes in women diagnosed with GDM [22] and evidence that lifestyle changes can improve outcomes [23], there is a paucity of reports on screening, and low rates have been reported [24,25]. The current National Institute for Health and Care Excellence (NICE) guidelines recommend screening for type 2 diabetes (between 6 and 13 weeks postpartum and an annual glycated hemoglobin [HbA1c] test) and lifestyle changes (weight control, diet, and exercise) for women diagnosed with GDM [26]. There is no recommendation to screen, identify, and actively manage cardiovascular risk factors (including hypertension, dyslipidemia, and smoking) in women diagnosed with GDM in the postpartum period in the current 2015 NICE guidelines [26].

The aim of this current study is to examine the risk of cardiovascular disease in women previously diagnosed with GDM in a population that is representative of all women diagnosed with GDM in the United Kingdom (UK). In addition, the proportion of women assessed for cardiovascular risk factors in the first 3 years postpartum in primary care will be documented. The results are expected to assist general practice in identifying and targeting cardiovascular risk factors in a group of relatively young women at high risk of long-term metabolic and cardiovascular disorders.

## Methods

The study protocol was approved by the Scientific Review Committee (SRC Reference Number: 17THIN001) of the data provider, IQVIA.

### Research design

A retrospective cohort study design was used to compare long-term cardiometabolic outcomes in women diagnosed with GDM and randomly matched pregnant control women not diagnosed with GDM, utilizing The Health Improvement Network (THIN) database—following the pre-analysis study plan (S1 Text). This database captures electronically recorded medical records in primary care and is designed to encourage research and improve healthcare delivery in the UK [27]. Over 675 general practices contribute to the THIN database, which captures about 6% (3.6 million) of the total registered population [27], is representative of the age structure of the UK population, and is made up predominantly of a white British, Welch, and Irish population (94% in 1991 although decreasing to 86% by 2011) [28]. Patient information is entered into the Vision patient record software, which uses Read code data (version 2) [29], rather than the World Health Organization International Classification of Diseases designed for hospital records. The Vision software also captures all British National Formulary drug prescription records [30]. General practices that had electronic medical record software for at least 1 year and had an acceptable mortality recording for at least 12 months were included in the analyses to ensure data quality and that all important covariates were recorded.

### Study population

All records for women who became pregnant between 1 February 1990 and 15 May 2016 and were aged less than 50 years were accessed for possible inclusion in the study. Women diagnosed with GDM prior to delivery were identified and randomly matched with up to 4 pregnant control women without GDM by age and timing of entry of a code for pregnancy (up to 3 months).

The primary outcomes were the clinical diagnosis of coronary artery disease (ischemic heart disease [IHD]) and cerebrovascular disease (stroke or transient ischemic attack [TIA]). Secondary outcomes were cases of incident hypertension and type 2 diabetes. Outcomes were identified through clinical codes (S1 Data). Recording of diabetes, hypertension, and cardiovascular disease is considered accurate in UK primary care because there is a mandatory requirement for maintaining a register for these conditions, and incentive payments are made for identification and management of these cardiometabolic outcomes [31].

Women with a diagnosis of the outcome of interest prior to baseline (i.e., index date: at diagnosis of GDM for cases and confirmation of pregnancy for control women) were not included in the analysis for that outcome. All potential and available risk factors for all outcomes of interest (Townsend quintile, smoking, and body mass index [BMI]) and confounding variables (hypertension and lipid-lowering medication at baseline for IHD and stroke or TIA) were extracted for each of the women included in the study to adjust for confounding.

### Patient involvement

Patients were not involved in the design of this research project, in conducting the study, or in preparing results or reports. In acknowledgment of the patients and general practices that contributed information to the THIN dataset, the published paper will be circulated via IQVIA to all current practices that contribute to the dataset.

### Statistical analyses

Characteristics of women with GDM and matched control women in the cohort were reported using appropriate descriptive statistics (mean and median for continuous variables and proportions for categorical variables). Incidence rate ratios (IRRs) and 95% confidence intervals (CIs) were calculated using the Poisson regression model, offsetting the exposure for person-years of follow-up. Adjusted IRRs were constructed by including age, BMI, Townsend quintile (a measure of deprivation), and smoking status in the Poisson models for hypertension and diabetes outcomes. In addition to these covariates, baseline hypertension and lipid-lowering medication prescription were included in the models for cardiovascular disease outcomes. BMI (in kg/m^2^) was treated as a categorical variable and grouped into <25, 25 to 30, and >30 kg/m^2^, based on the World Health Organization BMI categories [32].

Missing data for BMI, Townsend quintile, and smoking were included in the regression model as a missing categorical variable. We did not include ethnicity in our primary analysis because of poor recording in the primary care setting (<50%). However, in a sensitivity analysis, we included the available recording of ethnicity along with a missing category in the model to assess its impact on findings. Statistical significance was set at 0.05. Cumulative incidence curves were generated utilizing the cumulative incidence function of the survival curves. In addition, we report on the proportions of women with GDM and control women who were screened in the subsequent 3 years postpartum for smoking, BMI, diabetes, hypertension, and dyslipidemia. All analyses were conducted using STATA 14.0 [33].

## Results

**Fig 1:**
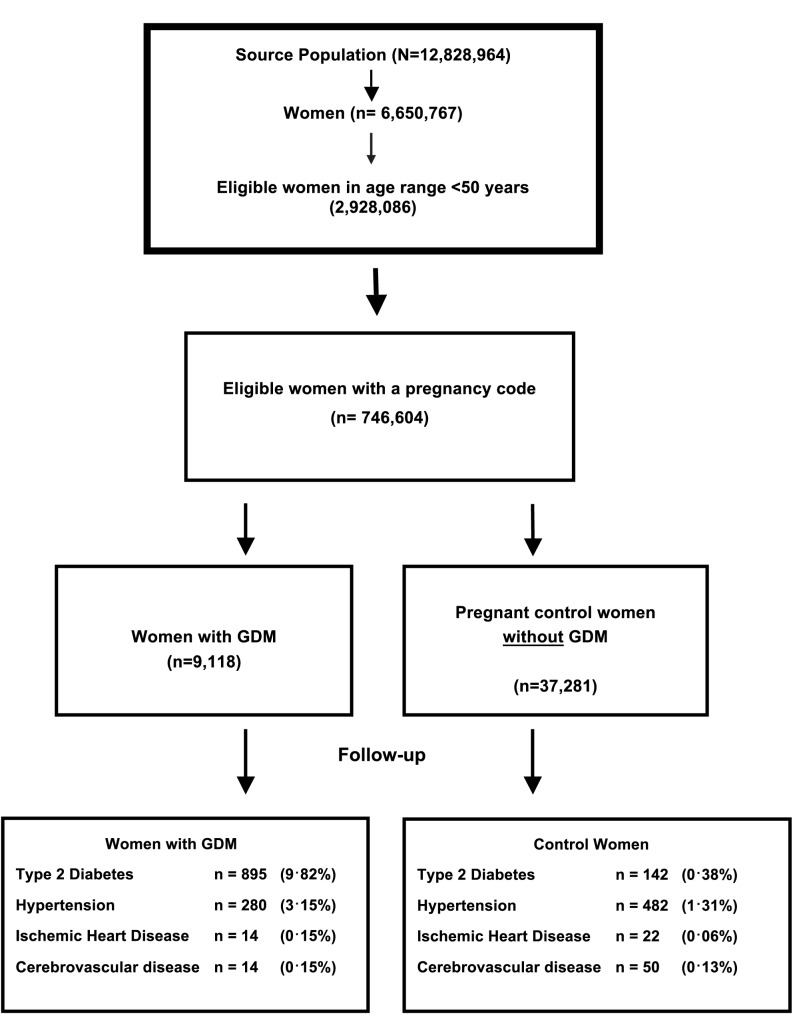
Flow diagram of the source population, women with gestation diabetes mellitus (GDM), and matched controls, including the proportion of women followed up in each group.

A total of 9,118 women with GDM (based on an electronic code entry for GDM) were identified in the dataset. Table 1 outlines the demographic characteristics of the women diagnosed with GDM compared with pregnant control women (matched by age and timing of pregnancy) at baseline. Mean age at the time of delivery was 33 years and ranged from 14 to 47 years. A significantly greater proportion of women with GDM compared with controls were from economically deprived areas (Townsend quintile 4 or 5), were overweight or obese (BMI ≥ 25 kg/m^2^, 63% compared with 35%), and had been diagnosed with hypertension (including 2.5% prior to pregnancy), but women with GDM were less likely to be current smokers (16% versus 19%). The follow-up period varied from less than 1 to 25 years (median 2.9 years). There was a high proportion of SA women (17.1%) among those with a recording for ethnicity in the GDM cohort. Fig 1 outlines the sampling frame for the total number of pregnant women, those diagnosed with GDM, and control women matched by age and time of pregnancy.

**Table 1:** Participant baseline characteristics.

Characteristic	Women with GDM	Control women	*p-*Value
**Population *n***	9,118	37,281	
**Age (years)**			
Mean (SD)	33 (5.4)	33 (5.4)	0.22
Median (IQR)	33 (29–37)	33 (29–37)	0.21
**BMI category**			
<25 kg/m^2^	2,338 (26)	18,514 (50)	<0.001
25–30 kg/m^2^	2,220 (24)	7,943 (21)	
>30 kg/m^2^	3,548 (39)	5,217 (14)	
Missing or implausible value	1,012 (11)	5,607 (15)	
**Townsend quintile**			
1	1,658 (18)	7,698 (21)	<0.001
2	1,494 (16)	7,076 (19)	
3	1,913 (21)	7,595 (20)	
4	1,863 (20)	6,877 (18)	
5	1,505 (17)	4,805 (13)	
Missing value	685 (8)	3,230 (9)	
**Ethnicity**			
White	2,802 (30.7)	14,930 (40.0)	<0.001
South Asian	687 (7.5)	1,219 (3.3)	
Afro-Caribbean	237 (2.6)	828 (2.2)	
Other	284 (3.1)	900 (2.4)	
Missing value	5,108 (56.0)	19,404 (52.0)	
**Smoker**			
No	5,696 (62)	22,470 (60)	<0.001
Discontinued	1,738 (19)	6,445 (17)	
Yes	1,480 (16)	7,190 (19)	
Missing value	204 (2)	1,176 (3)	
**Baseline medical conditions**			
Hypertension	232 (2.54)	431 (1.16)	<0.001
Ischemic heart disease	6 (0.07)	4 (0.01)	0.001
Stroke or TIA	12 (0.13)	39 (0.10)	0.486

Values are *n* (percent) unless otherwise indicated. *p-*Values show significance of variation in percentages in subgroups, from the *t* test for age and chi-squared test for all other variables.

BMI, body mass index; GDM, gestational diabetes mellitus; TIA, transient ischemic attack.

Table 2 shows that women diagnosed with GDM were over 20 times more likely to develop type 2 diabetes (IRR = 21.96; 95% CI 18.31–26.34; *p-*value < 0.001) and had almost a 2-fold higher risk of developing hypertension (IRR = 1.85; 95% CI 1.59–2.16; *p-*value < 0.001) after adjusting for age, Townsend quintile, BMI, and smoking compared with control women. Further, after controlling for baseline lipid-lowering medication and hypertension in addition to the above covariates, women with GDM were more than 2.5 times more likely to develop IHD (IRR = 2.78; 95% CI 1.37–5.66; *p-*value = 0.005), but no increase in risk was found for cerebrovascular disease (IRR = 0.95; 95% CI 0.51–1.77; *p-*value = 0.87). Of the 14 women with GDM who developed IHD, only 5 also developed type 2 diabetes in the postpartum period, suggesting that the risk of cardiovascular disease is not always mediated through type 2 diabetes.

**Table 2:** Women with gestational diabetes mellitus (*n =* 9,118) who developed type 2 diabetes, hypertension, ischemic heart disease, and cerebrovascular disease in the postpartum period compared with control women (*n =* 37,281) who remained normoglycemic during pregnancy.

Value	Diabetes		Hypertension		Ischemic heart disease		Stroke or TIA	
	Cases	Controls	Cases	Controls	Cases	Controls	Cases	Controls
**Total *n***	9,118	37,281	8,886	36,850	9,112	37,277	9,106	37,242
**Incidence *n* (%)**	895 (9.82)	142 (0.38)	280 (3.15)	482 (1.31)	14 (0.15)	22 (0.06)	14 (0.15)	50 (0.13)
**Person-years of follow-up**	35,715	157,600	37,327	153,769	39,583	157,958	39,601	157,764
**Incidence rate per 1,000 person-years**	25.06	0.90	7.50	3.13	0.35	0.14	0.35	0.32
**Incidence rate ratio (95% CI)**	27.81 (23.30–33.20)		2.39 (2.07–2.77)		2.54 (1.30–4.96)		1.12 (0.62–2.02)	
*p-*Value	<0.001		<0.001		0.006		0.718	
**Adjusted incidence rate ratio (95% CI)**	21.96 (18.31–26.34)*		1.85 (1.59–2.16)*		2.78 (1.37–5.66)^†^		0.95 (0.51–1.77)^†^	
*p-*Value	<0.001		<0.001		0.005		0.87	

*Adjusted for age, Townsend quintile, BMI, and smoking.

^†^Adjusted for age, Townsend quintile, BMI, smoking, prescribed lipid-lowering medication, and hypertension.

BMI, body mass index; TIA, transient ischemic attack.

**Fig 2:**
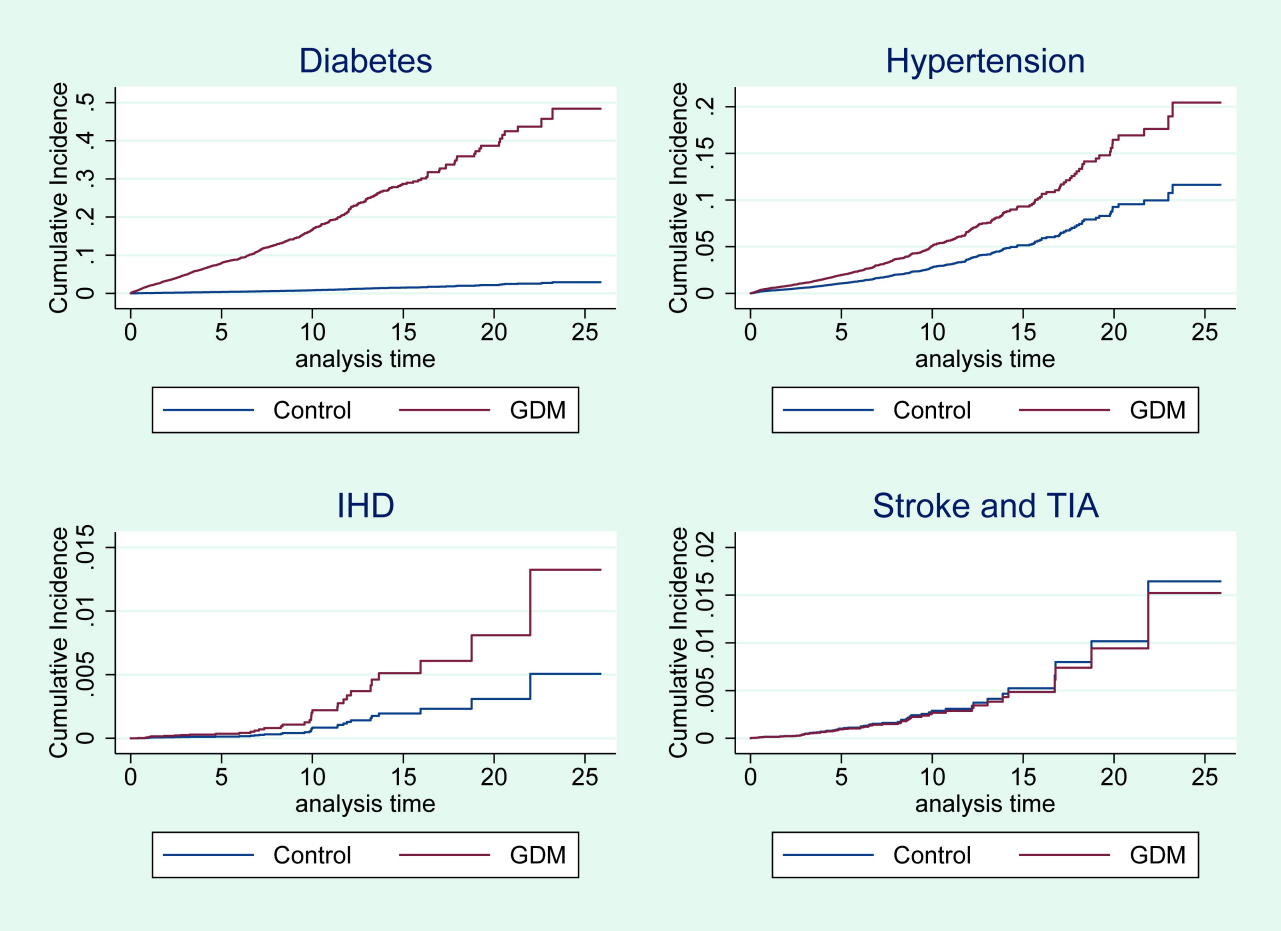
Cumulative incidence of diabetes, hypertension, IHD, and stroke or TIA for women with GDM and control women. The analyses for diabetes and hypertension are adjusted for age, Townsend quintile, BMI, and smoking. The analyses for IHD and stroke or TIA are adjusted for the above covariates, prescribed lipid-lowering medication, and hypertension. BMI, body mass index; GDM, gestational diabetes mellitus; IHD, ischemic heart disease; TIA, transient ischemic attack.

Fig 2 shows that the cumulative incidence of type 2 diabetes, hypertension, and IHD was higher for women with GDM compared with control women and that this difference persisted throughout the 25-year study period. The increased risk was specific for type 2 diabetes, hypertension, and IHD but not for stroke or TIA.

The sensitivity analysis that included ethnicity in the model did not significantly alter the effect sizes for any of the outcomes: IRR for type 2 diabetes = 21.08 (95% CI 17.57–25.30), IRR for hypertension = 1.82 (95% CI 1.56–2.13), IRR for IHD = 2.72 (95% CI 1.33–5.60), and IRR for stroke or TIA = 0.91 (95% CI 0.48–1.70). In an analysis restricted to women with GDM, SA women were twice as likely (IRR = 2.09; 95% CI 1.52–2.85) as white women to develop type 2 diabetes, and Afro-Caribbean (AC) women were 1.6 times more likely (IRR = 1.65; 95% CI 1.05–2.62). There was no increased risk for hypertension in AC women (IRR = 1.35; 95% CI 0.60–3.02) or SA women (IRR = 0.85; 95% CI 0.41–1.77) with GDM compared with white women with GDM. Ethnic subgroup analysis showed that white, AC, and SA women with GDM were at higher risk of developing type 2 diabetes than women without GDM, with IRR (95% CI) values of 35.2 (20.0–58.5), 22.15 (6.42–76.4) and 15.40 (6.54–36.25), respectively. Similar analyses for other outcomes were not possible due to the small number of outcomes among the relatively small number of women from the 2 minority ethnic groups.

Medical records for women with GDM showed that only 58% had some form of glycemic measurement in the first year following delivery (Table 3). Although 62% of women with GDM were tested after 1 January 2010, per the original 2008 NICE guideline publication recommending screening for type 2 diabetes [34], this proportion was not markedly different from the 53% tested prior to 2010. In the second and third year following delivery, the proportion with glycemic measurement decreased to less than 40%, and 24% of women did not have any glycemic measurement in the first 3 years postpartum. No difference was noted in the proportion of women with GDM who had blood pressure measured and recorded prior to and from 2010, with about 80% in the first year following delivery and declining to around 50% in the subsequent 2 years. BMI was recorded for almost half of women with GDM in the first year following delivery, decreasing to about a third in the subsequent 2 years, and did not change following the original guideline publication. Smoking status was recorded for 46% of women in the first year following delivery, and although this proportion declined in the second and third year, 73% had at least 1 record over this 3-year period. Lipid profiles were the most poorly recorded for women with GDM in the 3 years following delivery. Only 28% and 23% of women had any serum cholesterol or triglyceride level, respectively, recorded in this 3-year period.

**Table 3:** Follow-up screening for women with gestational diabetes mellitus (GDM, *n =* 9,118) and control women (*n =* 37,281).

Follow-up screening*	GDM before 2010, *n* (%)	GDM from 2010, *n* (%)	All GDM, *n* (%)	Control, *n* (%)
**Body mass index**				
Between 0 and 1 year postpartum	1,449 (46)	1,904 (48)	3,353 (48)	9,510 (33)
Between 1 and 2 years postpartum	993 (35)	922 (35)	1,915 (35)	6,574 (29)
Between 2 and 3 years postpartum	830 (32)	566 (35)	1,396 (33)	5,147 (28)
**Blood tests for diabetes**^†^				
Between 0 and 1 year postpartum	1,648 (53)	2,428 (62)	4,076 (58)	4,974 (17)
Between 1 and 2 years postpartum	993 (35)	1,111 (42)	2,104 (38)	3,286 (14)
Between 2 and 3 years postpartum	832 (32)	677 (42)	1,509 (36)	2,803 (15)
**Blood pressure**				
Between 0 and 1 year postpartum	2,455 (79)	3,195 (81)	5,650 (80)	18,933 (65)
Between 1 and 2 years postpartum	1,439 (51)	1,269 (48)	2,708 (50)	11,196 (49)
Between 2 and 3 years postpartum	1,251 (48)	781 (48)	2,032 (48)	8,825 (49)
**Smoking**				
Between 0 and 1 year postpartum	1,316 (42)	1,948 (49)	3,264 (46)	11,219 (39)
Between 1 and 2 years postpartum	1,028 (36)	961 (37)	1,989 (36)	8,320 (36)
Between 2 and 3 years postpartum	916 (35)	583 (36)	1,499 (35)	6,611 (36)
**All tests for cholesterol**^**#**^				
Between 0 and 1 year postpartum	320 (10)	476 (12)	796 (11)	1,217 (4)
Between 1 and 2 years postpartum	344 (12)	360 (14)	704 (13)	1,288 (6)
Between 2 and 3 years postpartum	302 (12)	246 (15)	548 (13)	1,222 (7)

*Eligible for follow-up in year 1: GDM *n =* 7,063 and control *n =* 28,968; year 2: GDM *n =* 5,474 and control *n =* 22,913; and year 3: GDM *n =* 4,239 and control *n =* 18,189.

^†^Includes all women who underwent at least 1 glycemic measurement (HbA1c, serum glucose, fasting glucose, or glucose tolerance test).

^#^Includes all women who underwent at least 1 lipid measurement (total cholesterol, high-density lipoprotein cholesterol, low-density lipoprotein cholesterol, or serum triglyceride level).

In the control population, surveillance for risk factors in the postpartum period was comparatively lower than for women diagnosed with GDM across all assessments in year 1, and this difference persisted for type 2 diabetes and lipid measurements in years 2 and 3. In contrast, the proportion assessed for hypertension and smoking in years 2 and 3 did not differ between control women and women diagnosed with GDM. In a sensitivity analysis limited to patients who did not develop hypertension in the first year and were followed-up for more than 1 year, the increased risk for hypertension persisted (hazard ratio [HR] = 1.87; 95% CI 1.57–2.24), suggesting that surveillance bias did not affect this outcome.

## Discussion

This is, to our knowledge, the first large population-based study in the UK that reports on the increased risk of cardiovascular disease in women diagnosed with GDM, and quantifies the high incidence of type 2 diabetes and hypertension for these women in the postpartum period. Women diagnosed with GDM were over 20 times more likely to develop type 2 diabetes, had almost twice the risk of developing hypertension, and were 2.8 times more likely to develop IHD in the postpartum period compared with control women. The increased risk persisted throughout the 25-year follow-up period. Despite the high risk of developing type 2 diabetes and cardiovascular disease, postpartum screening was poor, with less than 60% of women undergoing any type of screening test for diabetes in the 12 months following delivery and with the proportion declining to below 40% in the second year. Further, only half of the women with GDM had their blood pressure recorded in the second year following delivery. About a third had smoking status recorded, and very few women had lipids recorded, in the third year postpartum. The only improvement noted following publication of the 2008 NICE guidelines [34] was in any form of measurement for glycemia, and this improvement was only moderate.

Our findings are broadly consistent with the French study utilizing hospital records, the Canadian study utilizing primary care records, and the Nurses’ Health Study II using self-reported diagnosis of GDM. The French study reported a higher adjusted odds ratio (OR) for hypertension (2.72; 95% CI 2.58–2.88) than our study’s IRR, but a lower adjusted OR for cardiovascular outcomes that included angina pectoris (1.68; 95% CI 1.29–2.20) and myocardial infarction (1.92; 95% CI 1.36–2.71). Similar to our finding, the French study reported no effect for stroke [18]. The Canadian study reported an attenuated effect (HR = 2.09; 95% CI 1.19–3.67) for the development of coronary artery disease [17]. A significant but lower effect size was reported for the Nurses’ Health Study II for myocardial infarction (HR = 1.56; 95% CI 1.09–2.23) compared with our study, and no effect for stroke (HR = 1.22; 95% CI 0.80–1.86). In addition, a large Swedish case–control study also utilizing hospital records reported an increased risk of cardiovascular events (OR = 1.51; 95% CI 1.07–2.14) and hypertension (OR = 5.10; 95% CI 3.18–8.18) for women previously diagnosed with GDM compared with control women who had not had a cardiovascular event prior to the matched pregnancy and without a history of GDM [35]. Smaller studies have also shown associations between GDM and hypertension and cardiovascular disease in women with a family history of type 2 diabetes [36], and a higher risk of hypertension for Hispanic compared with white North American women diagnosed with GDM [37].

The increased incidence of type 2 diabetes for women with GDM found in our study is consistent with previous studies. Despite this, the incidence in this study was far higher than that reported in a review of observational studies (relative risk = 7.43, 95% CI 4.79–11.51) that also reported an increased risk after 5 years compared with the first 5 years postpartum [16]. However, the high incidence of type 2 diabetes in the first few years postpartum found in this current study was similar to findings in an older review that reported a higher cumulative incidence in the first 5 years postpartum than in subsequent years, after adjusting for cohort retention [15].

Historically, follow-up screening for type 2 diabetes in women diagnosed with GDM is poor in the postpartum period [38]. This study shows that fewer than 60% of women with diabetes in pregnancy were screened for type 2 diabetes, and far fewer had smoking status or lipids recorded, in the first year following delivery. Blood pressure recordings also decreased from 80% in the first year to 50% in the second year following delivery. The current 2015 NICE guidelines recommend annual screening for diabetes and lifestyle advice on “weight control, diet and exercise” for all women diagnosed with GDM [26]. However, there is no recommendation for screening and management of cardiovascular risk factors such as hypertension, dyslipidemia, or tobacco use following delivery in this group of high-risk women. In addition to enhancing early identification of type 2 diabetes, targeting this group also ensures that women know of their increased risk, which is important to them [39], and presents an opportunity to provide education and support for the lifestyle changes required to improve long-term outcomes [9,11], although there is currently a lack of evidence on exactly how to achieve this [40].

The study has several limitations. Our study captured women with GDM only if the condition was documented in the primary care medical records. Our estimates suggest we may have only captured around 49% of women with GDM in the THIN database (S1 Table). Selective documentation of women with more severe GDM may have resulted in an overestimation of the effect size, while any women with GDM misclassified in the control population may have resulted in an underestimation of the effect size. Though overall our findings are consistent with previous studies, the effect estimates for diabetes and IHD were higher than the estimates observed in other studies, while effect estimates were lower for hypertension and similar for stroke. Moreover, women with GDM may have underreported the use of tobacco during pregnancy as they were more likely to be overweight and to live in economically deprived areas, both of which are strongly associated with tobacco use in pregnancy [41]. A previous review of observational studies also found no association between smoking and GDM [42].

The timing and frequency of diabetes-related screening tests during and after pregnancy varied, potentially leading to a nondifferential error, with more women with GDM being diagnosed with diabetes and hypertension. Higher frequency of blood pressure measurements in the GDM population was noted only in year 1 of follow-up. When limiting our analysis to women without hypertension in the first year postpartum and with follow-up recordings beyond 1 year, the effect sizes remained the same for hypertension. Surveillance bias is less likely to affect outcomes that are symptomatic such as IHD and stroke. Further, there is limited information on baseline characteristics such as ethnicity and the number and order of pregnancies, limiting further in-depth analyses on factors that modify each of the outcomes. In particular, not sufficiently controlling for ethnicity might have resulted in an overestimation of the risk of type 2 diabetes in women with previous GDM, but this overestimation is likely to be small considering that the majority of women in the UK over the study period were white [28].

Additional limitations include the short median follow-up period, resulting in few women diagnosed with cardiovascular disease, and possible misclassification of outcome data related to the Read codes, which, although are ideally suited for general practice, are not always accurate. However, the outcomes are expected to be reported as part of the Quality and Outcomes Framework and have been shown to be reliable [43].

Despite these limitations, this study is, to our knowledge, the first UK and the largest population-based study of women with GDM utilizing primary care records to report on incidence of cardiovascular disease not requiring a hospital admission [17]. The findings add an important insight into the trajectory of the development of type 2 diabetes, hypertension, and cardiovascular disease in the early and later postpartum periods. Findings are consistent with previous reports on the risk of developing type 2 diabetes and cardiovascular disease. Furthermore, the findings report on a large population and identify an at-risk group of relatively young women ideally suited for targeting of risk factor management to improve long-term metabolic and cardiovascular outcomes. Targeting these high-risk women may also provide better value for money for prevention programs, as they are already known to general practice. While the value of preventing cardiovascular outcomes requires further studies, there is some evidence that targeting this subgroup of women may yield benefits in reducing conversion to type 2 diabetes [44].

### Conclusion

Results showed that women diagnosed with GDM were significantly more likely to develop type 2 diabetes, hypertension, and IHD at a relatively young age compared with women without a previous diagnosis of GDM. The risk was greatest for type 2 diabetes in the first year following delivery and persisted for 25 years. Follow-up screening for type 2 diabetes was poor, with less than 60% of women with GDM undergoing screening in the first year following delivery, and the proportion decreased to less than 40% by the second year. Guideline recommendations for screening and management of hypertension, lipids, and smoking cessation are lacking and need to be reviewed.

## Supporting information

S1 Data Participant selection, blood tests, and Read codes. (DOCX)

S1 STROBE Statement (DOCX)

S1 Table Tabulated calculations estimating the total number of women in the THIN dataset expected to have gestational diabetes mellitus during the study period. (DOCX)

S1 Text Analysis plan. (DOCX)

## Abbreviations

AC: Afro-Caribbean
BMI: body mass index
CI: confidence interval
GDM: gestational diabetes mellitus
HR: hazard ratio
IHD: ischemic heart disease
IRR: incidence rate ratio
NICE: National Institute for Health and Care Excellence
OR: odds ratio
SA: South Asian
THIN: The Health Improvement Network
TIA: transient ischemic attack
