# Correction: Increased risk of ischemic heart disease, hypertension, and type 2 diabetes in women with previous gestational diabetes mellitus, a target group in general practice for preventive interventions: A population-based cohort study

**DOI:** 10.1371/journal.pmed.1002881

**Published:** 2019-07-18

**Authors:** Barbara Daly, Konstantinos A. Toulis, Neil Thomas, Krishna Gokhale, James Martin, Jonathan Webber, Deepi Keerthy, Kate Jolly, Ponnusamy Saravanan, Krishnarajah Nirantharakumar

The authors misinterpreted a result within Reference 35 (Fadl et al) and therefore reported it incorrectly in the text of their article. The sentence that includes the reference, the sixth sentence within the second paragraph of the Discussion section, should read:

“In addition, a large Swedish case-control study also utilizing hospital records reported an association between cardiovascular events [OR = 1·51; 95% CI 1·07–2·14] and women previously diagnosed with GDM [35].”
