# Supplementary material for: Increased risk of ischemic heart disease, hypertension, and type 2 diabetes in women with previous gestational diabetes mellitus, a target group in general practice for preventive interventions: A population-based cohort study
Source: PLoS Med. 2018 Jan 16;15(1):e1002488. doi: 10.1371/journal.pmed.1002488 (PMC5770032; doi:10.1371/journal.pmed.1002488)
Supplement: S1 Data — (DOCX) [file pmed.1002488.s001.docx]

**S3 Participant selection, blood tests and Read codes**

**Contents**

[Section 1. Visual presentation of participant selection process 2](#_Toc485378876)

[Section 2. Details of blood tests carried out for glycaemic measurement and lipid measurement 3](#_Toc485378877)

[Section 3. Read codes used to identify GDM and outcomes 4](#_Toc485378878)

### Section 1. Visual presentation of participant selection process

Identification of exposed group (GDM) and their pregnant controls (without GDM) matched 1:4 by age and date of pregnancy code


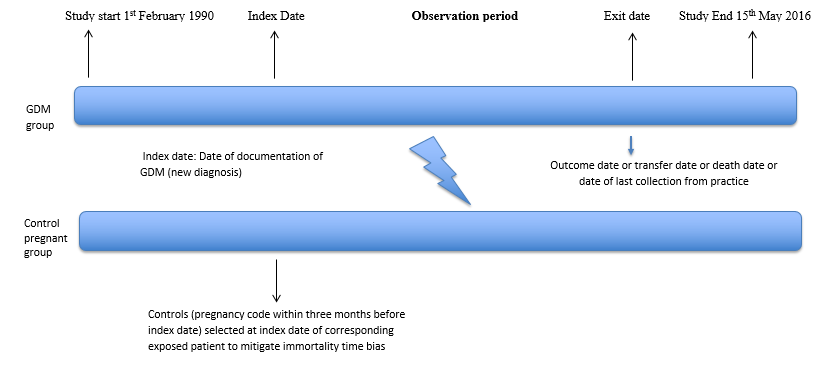


* To ensure good quality data, a patient is eligible to take part one year after the latest of the following dates: 1) registration in the practice (registration date); 2) introduction

of Electronic Medical Record; and 3) Acceptable Mortality Recording (AMR) date. AMR is an indicator when practices started to record information consistently and in a

timely manner.[1,2] One year latent period is applied to ensure there was sufficient time to record all important covariates. Figure 1: Visual presentation of participant selection process

**Figure 1. Visual presentation of participant selection process**

### Section 2. Blood tests carried out for glycaemic measurement and lipid measurement

### Table 1. Details of blood tests carried out for glycaemic measurement and lipid measurement

|  | **Before 2010** | **After 2010** | **All women** |
| --- | --- | --- | --- |
| **All blood tests for diabetes** | | | |
| ***between baseline and 1 year*** | 1648 (52.8%) | 2428 (61.6%) | 4076 (57.7%) |
| ***between 1-2 years*** | 993 (34.8%) | 1111 (42.4%) | 2104 (38.4%) |
| ***between 2-3 years*** | 832 (31.8%) | 677 (41.7%) | 1509 (35.6%) |
| **HbA1c** | | | |
| ***between baseline and 1 year*** | 554 (17.7%) | 988 (25.1%) | 1542 (21.8%) |
| ***between 1-2 years*** | 208 (7.3%) | 454 (17.3%) | 662 (12.1%) |
| ***between 2-3 years*** | 165 (6.3%) | 278 (17.1%) | 443 (10.5%) |
| **Random Blood Glucose** | | | |
| ***between baseline and 1 year*** | 1003 (32.1%) | 1345 (34.1%) | 2348 (33.2%) |
| ***between 1-2 years*** | 681 (23.9%) | 667 (25.4%) | 1348 (24.6%) |
| ***between 2-3 years*** | 576 (22.0%) | 392 (24.1%) | 968 (22.8%) |
| **Fasting glucose** | | | |
| ***between baseline and 1 year*** | 502 (16.1%) | 929 (23.6%) | 1431 (20.3%) |
| ***between 1-2 years*** | 322 (11.3%) | 391 (14.9%) | 713 (13.0%) |
| ***between 2-3 years*** | 277 (10.6%) | 236 (14.5%) | 513 (12.1%) |
| **Glucose tolerance Test** | | | |
| ***between baseline and 1 year*** | 575 (18.4%) | 543 (13.8%) | 1118 (15.8%) |
| ***between 1-2 years*** | 134 (4.7%) | 63 (2.4%) | 197 (3.6%) |
| ***between 2-3 years*** | 105 (4.0%) | 44 (2.7%) | 149 (3.5%) |
| **All test for Lipids** | | | |
| ***between baseline and 1 year*** | 320 (10.2%) | 476 (12.1%) | 796 (11.3%) |
| ***between 1-2 years*** | 344 (12.1%) | 360 (13.7%) | 704 (12.9%) |
| ***between 2-3 years*** | 302 (11.6%) | 246 (15.1%) | 548 (12.9%) |
| **Cholesterol** | | | |
| ***between baseline and 1 year*** | 318 (10.2%) | 473 (12.0%) | 791 (11.2%) |
| ***between 1-2 years*** | 342 (7.8%) | 360 (13.7%) | 702 (12.8%) |
| ***between 2-3 years*** | 301 (11.5%) | 246 (15.1%) | 547 (12.9%) |
| **Triglycerides** | | | |
| ***between baseline and 1 year*** | 243 (7.8%) | 383 (9.7%) | 626 (8.9%) |
| ***between 1-2 years*** | 280 (9.8%) | 299 (11.4%) | 579 (10.6%) |
| ***between 2-3 years*** | 251 (9.6%) | 202 (12.4%) | 453 (10.7%) |
| **High Density Lipoprotein** | | | |
| ***between baseline and 1 year*** | 249 (8.0%) | 425 (10.8%) | 674 (9.5%) |
| ***between 1-2 years*** | 269 (9.4%) | 331 (12.6%) | 600 (11.0%) |
| ***between 2-3 years*** | 266 (10.2%) | 226 (13.9%) | 492 (11.6%) |
| **Low Density Lipoprotein** | | | |
| ***between baseline and 1 year*** | 214 (6.9%) | 371 (9.4%) | 585 (8.3%) |
| ***between 1-2 years*** | 249 (8.7%) | 290 (11.1%) | 539 (9.8%) |
| ***between 2-3 years*** | 234 (9.0%) | 195 (12.0%) | 429 (10.1%) |

### Section 3. Read codes used to identify GDM and outcomes

- System based grouping (C10 for diabetes etc.)
- Use of flags in prioritising codes
- Read codes cited in previous studies
- Read codes listed in [www.clinicalcodes.org](http://www.clinicalcodes.org)
- Review of Read codes used in QOF
- Applying search for key terms describing a variable
- Discussion within the team
- Discussion with a GP
- Discussion with relevant clinical experts
- Discussion within the team (Second round)

Figure 1. Systematic process of identifying Read codes

**Table 1. Read Codes used for diagnosis of gestation diabetes mellitus (GDM)**

| **No.** | **Read Code** | **Description** | **Observed Numbers in final cohort** |
| --- | --- | --- | --- |
|  | L180811 | Gestational diabetes mellitus | 8,383 |
|  | L180900 | Gestational diabetes mellitus | 735 |
|  | ZC2CB00 | Dietary advice for gestational diabetes | 0 |
|  | 66Ay.00 | Gestational diabetes mellitus annual review | 0 |
|  | L180800 | Diabetes mellitus arising in pregnancy | 0 |

**Table 2. Read codes for diabetes outcomes**

| **Clinical code** | **Description*** |
| --- | --- |
| C10..00 | Diabetes mellitus |
| C100.00 | Diabetes mellitus with no mention of complication |
| C100000 | Diabetes mellitus, juvenile type, no mention of complication |
| C100011 | Insulin dependent diabetes mellitus |
| C100100 | Diabetes mellitus, adult onset, no mention of complication |
| C100111 | Maturity onset diabetes |
| C100112 | Non-insulin dependent diabetes mellitus |
| C100z00 | Diabetes mellitus NOS with no mention of complication |
| C101.00 | Diabetes mellitus with ketoacidosis |
| C101000 | Diabetes mellitus, juvenile type, with ketoacidosis |
| C101100 | Diabetes mellitus, adult onset, with ketoacidosis |
| C101y00 | Other specified diabetes mellitus with ketoacidosis |
| C101z00 | Diabetes mellitus NOS with ketoacidosis |
| C102.00 | Diabetes mellitus with hyperosmolar coma |
| C102000 | Diabetes mellitus, juvenile type, with hyperosmolar coma |
| C102100 | Diabetes mellitus, adult onset, with hyperosmolar coma |
| C102z00 | Diabetes mellitus NOS with hyperosmolar coma |
| C103.00 | Diabetes mellitus with ketoacidotic coma |
| C103000 | Diabetes mellitus, juvenile type, with ketoacidotic coma |
| C103100 | Diabetes mellitus, adult onset, with ketoacidotic coma |
| C103y00 | Other specified diabetes mellitus with coma |
| C103z00 | Diabetes mellitus NOS with ketoacidotic coma |
| C104.00 | Diabetes mellitus with renal manifestation |
| C104.11 | Diabetic nephropathy |
| C104000 | Diabetes mellitus, juvenile type, with renal manifestation |
| C104100 | Diabetes mellitus, adult onset, with renal manifestation |
| C104y00 | Other specified diabetes mellitus with renal complications |
| C104z00 | Diabetes mellitus with nephropathy NOS |
| C105.00 | Diabetes mellitus with ophthalmic manifestation |
| C105000 | Diabetes mellitus, juvenile type, + ophthalmic manifestation |
| C105100 | Diabetes mellitus, adult onset, + ophthalmic manifestation |
| C105y00 | Other specified diabetes mellitus with ophthalmic complicatn |
| C105z00 | Diabetes mellitus NOS with ophthalmic manifestation |
| C106.00 | Diabetes mellitus with neurological manifestation |
| C106.11 | Diabetic amyotrophy |
| C106.12 | Diabetes mellitus with neuropathy |
| C106.13 | Diabetes mellitus with polyneuropathy |
| C106000 | Diabetes mellitus, juvenile, + neurological manifestation |
| C106100 | Diabetes mellitus, adult onset, + neurological manifestation |
| C106y00 | Other specified diabetes mellitus with neurological comps |
| C106z00 | Diabetes mellitus NOS with neurological manifestation |
| C109.00 | Non-insulin dependent diabetes mellitus |
| C109.11 | NIDDM - Non-insulin dependent diabetes mellitus |
| C109.12 | Type 2 diabetes mellitus |
| C109.13 | Type II diabetes mellitus |
| C109000 | Non-insulin-dependent diabetes mellitus with renal comps |
| C109011 | Type II diabetes mellitus with renal complications |
| C109012 | Type 2 diabetes mellitus with renal complications |
| C109100 | Non-insulin-dependent diabetes mellitus with ophthalm comps |
| C109111 | Type II diabetes mellitus with ophthalmic complications |
| C109112 | Type 2 diabetes mellitus with ophthalmic complications |
| C109200 | Non-insulin-dependent diabetes mellitus with neuro comps |
| C109211 | Type II diabetes mellitus with neurological complications |
| C109212 | Type 2 diabetes mellitus with neurological complications |
| C109300 | Non-insulin-dependent diabetes mellitus with multiple comps |
| C109311 | Type II diabetes mellitus with multiple complications |
| C109312 | Type 2 diabetes mellitus with multiple complications |
| C109400 | Non-insulin dependent diabetes mellitus with ulcer |
| C109411 | Type II diabetes mellitus with ulcer |
| C109412 | Type 2 diabetes mellitus with ulcer |
| C109600 | Non-insulin-dependent diabetes mellitus with retinopathy |
| C109611 | Type II diabetes mellitus with retinopathy |
| C109612 | Type 2 diabetes mellitus with retinopathy |
| C109700 | Non-insulin dependent diabetes mellitus - poor control |
| C109711 | Type II diabetes mellitus - poor control |
| C109712 | Type 2 diabetes mellitus - poor control |
| C109800 | Reaven's syndrome |
| C109900 | Non-insulin-dependent diabetes mellitus without complication |
| C109911 | Type II diabetes mellitus without complication |
| C109912 | Type 2 diabetes mellitus without complication |
| C109A00 | Non-insulin dependent diabetes mellitus with mononeuropathy |
| C109A11 | Type II diabetes mellitus with mononeuropathy |
| C109A12 | Type 2 diabetes mellitus with mononeuropathy |
| C109B00 | Non-insulin dependent diabetes mellitus with polyneuropathy |
| C109B11 | Type II diabetes mellitus with polyneuropathy |
| C109B12 | Type 2 diabetes mellitus with polyneuropathy |
| C109C00 | Non-insulin dependent diabetes mellitus with nephropathy |
| C109C11 | Type II diabetes mellitus with nephropathy |
| C109C12 | Type 2 diabetes mellitus with nephropathy |
| C109D00 | Non-insulin dependent diabetes mellitus with hypoglyca coma |
| C109D11 | Type II diabetes mellitus with hypoglycaemic coma |
| C109D12 | Type 2 diabetes mellitus with hypoglycaemic coma |
| C109E00 | Non-insulin depend diabetes mellitus with diabetic cataract |
| C109E11 | Type II diabetes mellitus with diabetic cataract |
| C109E12 | Type 2 diabetes mellitus with diabetic cataract |
| C109G00 | Non-insulin dependent diabetes mellitus with arthropathy |
| C109G11 | Type II diabetes mellitus with arthropathy |
| C109G12 | Type 2 diabetes mellitus with arthropathy |
| C109H00 | Non-insulin dependent d m with neuropathic arthropathy |
| C109H11 | Type II diabetes mellitus with neuropathic arthropathy |
| C109H12 | Type 2 diabetes mellitus with neuropathic arthropathy |
| C109J00 | Insulin treated Type 2 diabetes mellitus |
| C109J11 | Insulin treated non-insulin dependent diabetes mellitus |
| C109J12 | Insulin treated Type II diabetes mellitus |
| C109K00 | Hyperosmolar non-ketotic state in type 2 diabetes mellitus |
| C10A.00 | Malnutrition-related diabetes mellitus |
| C10A000 | Malnutrition-related diabetes mellitus with coma |
| C10A100 | Malnutrition-related diabetes mellitus with ketoacidosis |
| C10A200 | Malnutrition-related diabetes mellitus with renal complicatn |
| C10A300 | Malnutrit-related diabetes mellitus wth ophthalmic complicat |
| C10A400 | Malnutrition-related diabetes mellitus wth neuro complicatns |
| C10A600 | Malnutrition-related diabetes mellitus with multiple comps |
| C10A700 | Malnutrition-related diabetes mellitus without complications |
| C10AW00 | Malnutrit-related diabetes mellitus with unspec complics |
| C10AX00 | Malnutrit-relat diabetes mellitus with other spec comps |
| C10B.00 | Diabetes mellitus induced by steroids |
| C10B000 | Steroid induced diabetes mellitus without complication |
| C10C.00 | Diabetes mellitus autosomal dominant |
| C10C.11 | Maturity onset diabetes in youth |
| C10C.12 | Maturity onset diabetes in youth type 1 |
| C10D.00 | Diabetes mellitus autosomal dominant type 2 |
| C10D.11 | Maturity onset diabetes in youth type 2 |
| C10F.00 | Type 2 diabetes mellitus |
| C10F.11 | Type II diabetes mellitus |
| C10F000 | Type 2 diabetes mellitus with renal complications |
| C10F011 | Type II diabetes mellitus with renal complications |
| C10F100 | Type 2 diabetes mellitus with ophthalmic complications |
| C10F111 | Type II diabetes mellitus with ophthalmic complications |
| C10F200 | Type 2 diabetes mellitus with neurological complications |
| C10F211 | Type II diabetes mellitus with neurological complications |
| C10F300 | Type 2 diabetes mellitus with multiple complications |
| C10F311 | Type II diabetes mellitus with multiple complications |
| C10F400 | Type 2 diabetes mellitus with ulcer |
| C10F411 | Type II diabetes mellitus with ulcer |
| C10F600 | Type 2 diabetes mellitus with retinopathy |
| C10F611 | Type II diabetes mellitus with retinopathy |
| C10F700 | Type 2 diabetes mellitus - poor control |
| C10F711 | Type II diabetes mellitus - poor control |
| C10F800 | Reaven's syndrome |
| C10F811 | Metabolic syndrome X |
| C10F900 | Type 2 diabetes mellitus without complication |
| C10F911 | Type II diabetes mellitus without complication |
| C10FA00 | Type 2 diabetes mellitus with mononeuropathy |
| C10FA11 | Type II diabetes mellitus with mononeuropathy |
| C10FB00 | Type 2 diabetes mellitus with polyneuropathy |
| C10FB11 | Type II diabetes mellitus with polyneuropathy |
| C10FC00 | Type 2 diabetes mellitus with nephropathy |
| C10FC11 | Type II diabetes mellitus with nephropathy |
| C10FD00 | Type 2 diabetes mellitus with hypoglycaemic coma |
| C10FD11 | Type II diabetes mellitus with hypoglycaemic coma |
| C10FE00 | Type 2 diabetes mellitus with diabetic cataract |
| C10FE11 | Type II diabetes mellitus with diabetic cataract |
| C10FG00 | Type 2 diabetes mellitus with arthropathy |
| C10FG11 | Type II diabetes mellitus with arthropathy |
| C10FH00 | Type 2 diabetes mellitus with neuropathic arthropathy |
| C10FH11 | Type II diabetes mellitus with neuropathic arthropathy |
| C10FJ00 | Insulin treated Type 2 diabetes mellitus |
| C10FJ11 | Insulin treated Type II diabetes mellitus |
| C10FK00 | Hyperosmolar non-ketotic state in type 2 diabetes mellitus |
| C10FK11 | Hyperosmolar non-ketotic state in type II diabetes mellitus |
| C10FL00 | Type 2 diabetes mellitus with persistent proteinuria |
| C10FL11 | Type II diabetes mellitus with persistent proteinuria |
| C10FM00 | Type 2 diabetes mellitus with persistent microalbuminuria |
| C10FM11 | Type II diabetes mellitus with persistent microalbuminuria |
| C10FN00 | Type 2 diabetes mellitus with ketoacidosis |

**Table 3. Read codes for hypertension**

| **Clinical code** | **Description** |
| --- | --- |
| G2...00 | Hypertensive disease |
| G2...11 | BP - hypertensive disease |
| G20..00 | Essential hypertension |
| G20..11 | High blood pressure |
| G20..12 | Primary hypertension |
| G200.00 | Malignant essential hypertension |
| G201.00 | Benign essential hypertension |
| G202.00 | Systolic hypertension |
| G203.00 | Diastolic hypertension |
| G20z.00 | Essential hypertension NOS |
| G20z.11 | Hypertension NOS |
| G24..00 | Secondary hypertension |
| G240.00 | Secondary malignant hypertension |
| G240000 | Secondary malignant renovascular hypertension |
| G240z00 | Secondary malignant hypertension NOS |
| G241.00 | Secondary benign hypertension |
| G241000 | Secondary benign renovascular hypertension |
| G241z00 | Secondary benign hypertension NOS |
| G244.00 | Hypertension secondary to endocrine disorders |
| G24z.00 | Secondary hypertension NOS |
| G24z000 | Secondary renovascular hypertension NOS |
| G24z100 | Hypertension secondary to drug |
| G24zz00 | Secondary hypertension NOS |
| G25..00 | Stage 1 hypertension (NICE - Nat Ins for Hth Clin Excl 2011) |
| G25..11 | Stage 1 hypertension |
| G250.00 | Stage 1 hyperten (NICE 2011) without evidence end organ damage |
| G251.00 | Stage 1 hyperten (NICE 2011) with evidence end organ damage |
| G26..00 | Severe hypertension (Nat Inst for Health Clinical Ex 2011) |
| G26..11 | Severe hypertension |
| G27..00 | Hypertension resistant to drug therapy |
| G28..00 | Stage 2 hypertension (NICE - Nat Ins for Hth Clin Excl 2011) |
| Gyu2000 | [X]Other secondary hypertension |
| Gyu2100 | [X]Hypertension secondary to other renal disorders |

**Table 4. Read codes for ischaemic heart disease**

| **Clinical code** | **Description** |
| --- | --- |
| G3...00 | Ischaemic heart disease |
| G3...11 | Arteriosclerotic heart disease |
| G3...12 | Atherosclerotic heart disease |
| G3...13 | IHD - Ischaemic heart disease |
| G30..00 | Acute myocardial infarction |
| G30..11 | Attack - heart |
| G30..12 | Coronary thrombosis |
| G30..13 | Cardiac rupture following myocardial infarction (MI) |
| G30..14 | Heart attack |
| G30..15 | MI - acute myocardial infarction |
| G30..16 | Thrombosis - coronary |
| G30..17 | Silent myocardial infarction |
| G300.00 | Acute anterolateral infarction |
| G301.00 | Other specified anterior myocardial infarction |
| G301000 | Acute anteroapical infarction |
| G301100 | Acute anteroseptal infarction |
| G301z00 | Anterior myocardial infarction NOS |
| G302.00 | Acute inferolateral infarction |
| G303.00 | Acute inferoposterior infarction |
| G304.00 | Posterior myocardial infarction NOS |
| G305.00 | Lateral myocardial infarction NOS |
| G306.00 | True posterior myocardial infarction |
| G307.00 | Acute subendocardial infarction |
| G307000 | Acute non-Q wave infarction |
| G307100 | Acute non-ST segment elevation myocardial infarction |
| G308.00 | Inferior myocardial infarction NOS |
| G309.00 | Acute Q-wave infarct |
| G30A.00 | Mural thrombosis |
| G30B.00 | Acute posterolateral myocardial infarction |
| G30X.00 | Acute transmural myocardial infarction of unspecif site |
| G30X000 | Acute ST segment elevation myocardial infarction |
| G30y.00 | Other acute myocardial infarction |
| G30y000 | Acute atrial infarction |
| G30y100 | Acute papillary muscle infarction |
| G30y200 | Acute septal infarction |
| G30yz00 | Other acute myocardial infarction NOS |
| G30z.00 | Acute myocardial infarction NOS |
| G31..00 | Other acute and subacute ischaemic heart disease |
| G310.00 | Postmyocardial infarction syndrome |
| G310.11 | Dressler's syndrome |
| G311.00 | Preinfarction syndrome |
| G311.11 | Crescendo angina |
| G311.12 | Impending infarction |
| G311.13 | Unstable angina |
| G311.14 | Angina at rest |
| G311000 | Myocardial infarction aborted |
| G311011 | MI - myocardial infarction aborted |
| G311100 | Unstable angina |
| G311200 | Angina at rest |
| G311300 | Refractory angina |
| G311400 | Worsening angina |
| G311500 | Acute coronary syndrome |
| G311z00 | Preinfarction syndrome NOS |
| G312.00 | Coronary thrombosis not resulting in myocardial infarction |
| G31y.00 | Other acute and subacute ischaemic heart disease |
| G31y000 | Acute coronary insufficiency |
| G31y100 | Microinfarction of heart |
| G31y200 | Subendocardial ischaemia |
| G31y300 | Transient myocardial ischaemia |
| G31yz00 | Other acute and subacute ischaemic heart disease NOS |
| G32..00 | Old myocardial infarction |
| G32..11 | Healed myocardial infarction |
| G32..12 | Personal history of myocardial infarction |
| G33..00 | Angina pectoris |
| G330.00 | Angina decubitus |
| G330000 | Nocturnal angina |
| G330z00 | Angina decubitus NOS |
| G331.00 | Prinzmetal's angina |
| G331.11 | Variant angina pectoris |
| G332.00 | Coronary artery spasm |
| G33z.00 | Angina pectoris NOS |
| G33z000 | Status anginosus |
| G33z100 | Stenocardia |
| G33z200 | Syncope anginosa |
| G33z300 | Angina on effort |
| G33z400 | Ischaemic chest pain |
| G33z500 | Post infarct angina |
| G33z600 | New onset angina |
| G33z700 | Stable angina |
| G33zz00 | Angina pectoris NOS |
| G34..00 | Other chronic ischaemic heart disease |
| G340.00 | Coronary atherosclerosis |
| G340.11 | Triple vessel disease of the heart |
| G340.12 | Coronary artery disease |
| G340000 | Single coronary vessel disease |
| G340100 | Double coronary vessel disease |
| G341.00 | Aneurysm of heart |
| G341.11 | Cardiac aneurysm |
| G341000 | Ventricular cardiac aneurysm |
| G341100 | Other cardiac wall aneurysm |
| G341111 | Mural cardiac aneurysm |
| G341200 | Aneurysm of coronary vessels |
| G341300 | Acquired atrioventricular fistula of heart |
| G341z00 | Aneurysm of heart NOS |
| G342.00 | Atherosclerotic cardiovascular disease |
| G343.00 | Ischaemic cardiomyopathy |
| G344.00 | Silent myocardial ischaemia |
| G34y.00 | Other specified chronic ischaemic heart disease |
| G34y000 | Chronic coronary insufficiency |
| G34y100 | Chronic myocardial ischaemia |
| G34yz00 | Other specified chronic ischaemic heart disease NOS |
| G34z.00 | Other chronic ischaemic heart disease NOS |
| G34z000 | Asymptomatic coronary heart disease |
| G35..00 | Subsequent myocardial infarction |
| G350.00 | Subsequent myocardial infarction of anterior wall |
| G351.00 | Subsequent myocardial infarction of inferior wall |
| G353.00 | Subsequent myocardial infarction of other sites |
| G35X.00 | Subsequent myocardial infarction of unspecified site |
| G36..00 | Certain current complication follow acute myocardial infarct |
| G360.00 | Haemopericardium/current comp folow acut myocard infarct |
| G361.00 | Atrial septal defect/curr comp folow acut myocardal infarct |
| G362.00 | Ventric septal defect/curr comp fol acut myocardal infarctn |
| G363.00 | Ruptur cardiac wall w'out haemopericard/cur comp fol ac MI |
| G364.00 | Ruptur chordae tendinae/curr comp fol acute myocard infarct |
| G365.00 | Rupture papillary muscle/curr comp fol acute myocard infarct |
| G366.00 | Thrombosis atrium,auric append&vent/curr comp foll acute MI |
| G37..00 | Cardiac syndrome X |
| G38..00 | Postoperative myocardial infarction |
| G380.00 | Postoperative transmural myocardial infarction anterior wall |
| G381.00 | Postoperative transmural myocardial infarction inferior wall |
| G382.00 | Postoperative transmural myocardial infarction other sites |
| G383.00 | Postoperative transmural myocardial infarction unspec site |
| G384.00 | Postoperative subendocardial myocardial infarction |
| G38z.00 | Postoperative myocardial infarction, unspecified |
| G39..00 | Coronary microvascular disease |
| G3y..00 | Other specified ischaemic heart disease |
| G3z..00 | Ischaemic heart disease NOS |
| Gyu3.00 | [X]Ischaemic heart diseases |
| Gyu3000 | [X]Other forms of angina pectoris |
| Gyu3100 | [X]Other current complications following acute myocard infarct |
| Gyu3200 | [X]Other forms of acute ischaemic heart disease |
| Gyu3300 | [X]Other forms of chronic ischaemic heart disease |
| Gyu3400 | [X]Acute transmural myocardial infarction of unspecif site |
| Gyu3500 | [X]Subsequent myocardial infarction of other sites |
| Gyu3600 | [X]Subsequent myocardial infarction of unspecified site |

**Table 5. Read codes for stroke, and trans-ischaemic attack (cerebrovascular disease)**

| **Clinical code** | **Description** |
| --- | --- |
| G6...00 | Cerebrovascular disease |
| G60..00 | Subarachnoid haemorrhage |
| G600.00 | Ruptured berry aneurysm |
| G601.00 | Subarachnoid haemorrhage from carotid siphon and bifurcation |
| G602.00 | Subarachnoid haemorrhage from middle cerebral artery |
| G603.00 | Subarachnoid haemorrhage from anterior communicating artery |
| G604.00 | Subarachnoid haemorrhage from posterior communicating artery |
| G605.00 | Subarachnoid haemorrhage from basilar artery |
| G606.00 | Subarachnoid haemorrhage from vertebral artery |
| G60X.00 | Subarachnoid haemorrh from intracranial artery, unspecif |
| G60z.00 | Subarachnoid haemorrhage NOS |
| G61..00 | Intracerebral haemorrhage |
| G61..11 | CVA - cerebrovascular accid due to intracerebral haemorrhage |
| G61..12 | Stroke due to intracerebral haemorrhage |
| G610.00 | Cortical haemorrhage |
| G611.00 | Internal capsule haemorrhage |
| G612.00 | Basal nucleus haemorrhage |
| G613.00 | Cerebellar haemorrhage |
| G614.00 | Pontine haemorrhage |
| G615.00 | Bulbar haemorrhage |
| G616.00 | External capsule haemorrhage |
| G617.00 | Intracerebral haemorrhage, intraventricular |
| G618.00 | Intracerebral haemorrhage, multiple localized |
| G619.00 | Lobar cerebral haemorrhage |
| G61X.00 | Intracerebral haemorrhage in hemisphere, unspecified |
| G61X000 | Left sided intracerebral haemorrhage, unspecified |
| G61X100 | Right sided intracerebral haemorrhage, unspecified |
| G61z.00 | Intracerebral haemorrhage NOS |
| G62..00 | Other and unspecified intracranial haemorrhage |
| G620.00 | Extradural haemorrhage - nontraumatic |
| G621.00 | Subdural haemorrhage - nontraumatic |
| G622.00 | Subdural haematoma - nontraumatic |
| G623.00 | Subdural haemorrhage NOS |
| G62z.00 | Intracranial haemorrhage NOS |
| G63..00 | Precerebral arterial occlusion |
| G63..11 | Infarction - precerebral |
| G63..12 | Stenosis of precerebral arteries |
| G630.00 | Basilar artery occlusion |
| G631.00 | Carotid artery occlusion |
| G631.11 | Stenosis, carotid artery |
| G631.12 | Thrombosis, carotid artery |
| G632.00 | Vertebral artery occlusion |
| G633.00 | Multiple and bilateral precerebral arterial occlusion |
| G634.00 | Carotid artery stenosis |
| G63y.00 | Other precerebral artery occlusion |
| G63y000 | Cerebral infarct due to thrombosis of precerebral arteries |
| G63y100 | Cerebral infarction due to embolism of precerebral arteries |
| G63z.00 | Precerebral artery occlusion NOS |
| G64..00 | Cerebral arterial occlusion |
| G64..11 | CVA - cerebral artery occlusion |
| G64..12 | Infarction - cerebral |
| G64..13 | Stroke due to cerebral arterial occlusion |
| G640.00 | Cerebral thrombosis |
| G640000 | Cerebral infarction due to thrombosis of cerebral arteries |
| G641.00 | Cerebral embolism |
| G641.11 | Cerebral embolus |
| G641000 | Cerebral infarction due to embolism of cerebral arteries |
| G64z.00 | Cerebral infarction NOS |
| G64z.11 | Brainstem infarction NOS |
| G64z.12 | Cerebellar infarction |
| G64z000 | Brainstem infarction |
| G64z100 | Wallenberg syndrome |
| G64z111 | Lateral medullary syndrome |
| G64z200 | Left sided cerebral infarction |
| G64z300 | Right sided cerebral infarction |
| G64z400 | Infarction of basal ganglia |
| G65..00 | Transient cerebral ischaemia |
| G65..11 | Drop attack |
| G65..12 | Transient ischaemic attack |
| G65..13 | Vertebro-basilar insufficiency |
| G650.00 | Basilar artery syndrome |
| G650.11 | Insufficiency - basilar artery |
| G651.00 | Vertebral artery syndrome |
| G651000 | Vertebro-basilar artery syndrome |
| G652.00 | Subclavian steal syndrome |
| G653.00 | Carotid artery syndrome hemispheric |
| G654.00 | Multiple and bilateral precerebral artery syndromes |
| G655.00 | Transient global amnesia |
| G656.00 | Vertebrobasilar insufficiency |
| G657.00 | Carotid territory transient ischaemic attack |
| G65y.00 | Other transient cerebral ischaemia |
| G65z.00 | Transient cerebral ischaemia NOS |
| G65z000 | Impending cerebral ischaemia |
| G65z100 | Intermittent cerebral ischaemia |
| G65zz00 | Transient cerebral ischaemia NOS |
| G66..00 | Stroke and cerebrovascular accident unspecified |
| G66..11 | CVA unspecified |
| G66..12 | Stroke unspecified |
| G66..13 | CVA - Cerebrovascular accident unspecified |
| G660.00 | Middle cerebral artery syndrome |
| G661.00 | Anterior cerebral artery syndrome |
| G662.00 | Posterior cerebral artery syndrome |
| G663.00 | Brain stem stroke syndrome |
| G664.00 | Cerebellar stroke syndrome |
| G665.00 | Pure motor lacunar syndrome |
| G666.00 | Pure sensory lacunar syndrome |
| G667.00 | Left sided CVA |
| G668.00 | Right sided CVA |
| G669.00 | Cerebral palsy, not congenital or infantile, acute |
| G67..00 | Other cerebrovascular disease |
| G670.00 | Cerebral atherosclerosis |
| G670.11 | Precerebral atherosclerosis |
| G671.00 | Generalised ischaemic cerebrovascular disease NOS |
| G671000 | Acute cerebrovascular insufficiency NOS |
| G671100 | Chronic cerebral ischaemia |
| G671z00 | Generalised ischaemic cerebrovascular disease NOS |
| G672.00 | Hypertensive encephalopathy |
| G672.11 | Hypertensive crisis |
| G673.00 | Cerebral aneurysm, nonruptured |
| G673000 | Dissection of cerebral arteries, nonruptured |
| G673100 | Carotico-cavernous sinus fistula |
| G673200 | Carotid artery dissection |
| G673300 | Vertebral artery dissection |
| G674.00 | Cerebral arteritis |
| G674000 | Cerebral amyloid angiopathy |
| G675.00 | Moyamoya disease |
| G676.00 | Nonpyogenic venous sinus thrombosis |
| G676000 | Cereb infarct due cerebral venous thrombosis, nonpyogenic |
| G677.00 | Occlusion/stenosis cerebral arts not result cerebral infarct |
| G677000 | Occlusion and stenosis of middle cerebral artery |
| G677100 | Occlusion and stenosis of anterior cerebral artery |
| G677200 | Occlusion and stenosis of posterior cerebral artery |
| G677300 | Occlusion and stenosis of cerebellar arteries |
| G677400 | Occlusion+stenosis of multiple and bilat cerebral arteries |
| G678.00 | Cereb autosom dominant arteriop subcort infarcts leukoenceph |
| G679.00 | Small vessel cerebrovascular disease |
| G67A.00 | Cerebral vein thrombosis |
| G67B.00 | Reversible cerebral vasoconstriction syndrome |
| G67B.11 | Call-Fleming syndrome |
| G67y.00 | Other cerebrovascular disease OS |
| G67z.00 | Other cerebrovascular disease NOS |
| G68..00 | Late effects of cerebrovascular disease |
| G680.00 | Sequelae of subarachnoid haemorrhage |
| G681.00 | Sequelae of intracerebral haemorrhage |
| G682.00 | Sequelae of other nontraumatic intracranial haemorrhage |
| G683.00 | Sequelae of cerebral infarction |
| G68W.00 | Sequelae/other + unspecified cerebrovascular diseases |
| G68X.00 | Sequelae of stroke,not specfd as h'morrhage or infarction |
| G6y..00 | Other specified cerebrovascular disease |
| G6z..00 | Cerebrovascular disease NOS |
| Gyu6.00 | [X]Cerebrovascular diseases |
| Gyu6000 | [X]Subarachnoid haemorrhage from other intracranial arteries |
| Gyu6100 | [X]Other subarachnoid haemorrhage |
| Gyu6200 | [X]Other intracerebral haemorrhage |
| Gyu6300 | [X]Cerebrl infarctn due/unspcf occlusn or sten/cerebrl artrs |
| Gyu6400 | [X]Other cerebral infarction |
| Gyu6500 | [X]Occlusion and stenosis of other precerebral arteries |
| Gyu6600 | [X]Occlusion and stenosis of other cerebral arteries |
| Gyu6700 | [X]Other specified cerebrovascular diseases |
| Gyu6C00 | [X]Sequelae of stroke;not specfd as h'morrhage or infarction |
| Gyu6D00 | [X]Sequelae/other unspecified cerebrovascular diseases |
| Gyu6E00 | [X]Subarachnoid haemorrh from intracranial artery, unspecif |
| Gyu6F00 | [X]Intracerebral haemorrhage in hemisphere, unspecified |
| Gyu6G00 | [X]Cereb infarct due unsp occlus/stenos precerebr arteries |
| G6W..00 | Cereb infarct due unsp occlus/stenos precerebr arteries |
| G6X..00 | Cerebrl infarctn due/unspcf occlusn or sten/cerebrl artrs |

**References**

1. Maguire A, Blak BT, Thompson M. The importance of defining periods of complete mortality reporting for research using automated data from primary care. Pharmacoepidemiol Drug Saf. 2009;18(1):76-83. doi: 10.1002/pds.1688. PMID: 19065600

2. Horsfall L, Walters K, Petersen I. Identifying periods of acceptable computer usage in primary care research databases. Pharmacoepidemiol Drug Saf. 2013;22(1):64-9. doi: 10.1002/pds.3368. PMID: 23124958
