# Supplementary material for: Increased risk of ischemic heart disease, hypertension, and type 2 diabetes in women with previous gestational diabetes mellitus, a target group in general practice for preventive interventions: A population-based cohort study
Source: PLoS Med. 2018 Jan 16;15(1):e1002488. doi: 10.1371/journal.pmed.1002488 (PMC5770032; doi:10.1371/journal.pmed.1002488)
Supplement: S1 Table — (DOCX) [file pmed.1002488.s003.docx]

| **Year** | **UK population** | **Number of Live births** | **GDM Rate** | **Expected Mothers with GDM in UK** | **THIN population** | **Expected number of live births** | **Numbers captured in THIN** | **Expected GDM Pregnancies** | **Expected GDM Women** | **Observed GDM**  **Women** |
| --- | --- | --- | --- | --- | --- | --- | --- | --- | --- | --- |
| 1990 | 57,237,500 | 706,140 | 1.00 | 7,061 | 290325 | 3,582 | 3,224 | 36 |  | 3 |
| 1991 | 57,438,700 | 699,217 | 1.10 | 7,691 | 737180 | 8,974 | 8,077 | 99 |  | 8 |
| 1992 | 57,584,500 | 689,656 | 1.20 | 8,276 | 1142300 | 13,681 | 12,313 | 164 |  | 25 |
| 1993 | 57,713,900 | 673,467 | 1.30 | 8,755 | 1303416 | 15,210 | 13,689 | 198 |  | 26 |
| 1994 | 57,862,100 | 664,726 | 1.40 | 9,306 | 1463119 | 16,808 | 15,128 | 235 |  | 26 |
| 1995 | 58,024,800 | 648,138 | 1.50 | 9,722 | 1606957 | 17,950 | 16,155 | 269 |  | 19 |
| 1996 | 58,164,400 | 649,485 | 1.60 | 10,392 | 1790110 | 19,989 | 17,990 | 320 |  | 26 |
| 1997 | 58,314,200 | 643,095 | 1.70 | 10,933 | 1991942 | 21,967 | 19,771 | 373 |  | 40 |
| 1998 | 58,474,900 | 635,901 | 1.80 | 11,446 | 2311753 | 25,140 | 22,626 | 453 |  | 31 |
| 1999 | 58,684,400 | 621,872 | 1.90 | 11,816 | 2593972 | 27,488 | 24,739 | 522 |  | 66 |
| 2000 | 58,886,100 | 604,441 | 2.00 | 12,089 | 2913683 | 29,908 | 26,917 | 598 |  | 115 |
| 2001 | 59,113,016 | 594,634 | 2.10 | 12,487 | 3409158 | 34,294 | 30,864 | 720 |  | 171 |
| 2002 | 59,365,677 | 596,122 | 2.20 | 13,115 | 3707453 | 37,228 | 33,506 | 819 |  | 176 |
| 2003 | 59,636,662 | 621,469 | 2.30 | 14,294 | 3878976 | 40,423 | 36,380 | 930 |  | 230 |
| 2004 | 59,950,364 | 639,721 | 2.40 | 15,353 | 3988883 | 42,565 | 38,308 | 1,022 |  | 281 |
| 2005 | 60,413,276 | 645,835 | 2.50 | 16,146 | 4192022 | 44,814 | 40,333 | 1,120 |  | 327 |
| 2006 | 60,827,067 | 669,601 | 2.60 | 17,410 | 4340274 | 47,779 | 43,001 | 1,242 |  | 380 |
| 2007 | 61,319,075 | 690,013 | 2.70 | 18,630 | 4425593 | 49,800 | 44,820 | 1,345 |  | 429 |
| 2008 | 61,823,772 | 708,711 | 2.80 | 19,844 | 4514808 | 51,755 | 46,580 | 1,449 |  | 514 |
| 2009 | 62,260,486 | 706,248 | 2.90 | 20,481 | 4566859 | 51,804 | 46,623 | 1,502 |  | 628 |
| 2010 | 62,759,456 | 723,165 | 3.00 | 21,695 | 4460274 | 51,395 | 46,255 | 1,542 |  | 774 |
| 2011 | 63,285,145 | 723,913 | 3.20 | 23,165 | 4452683 | 50,934 | 45,840 | 1,630 |  | 818 |
| 2012 | 63,705,030 | 729,674 | 3.40 | 24,809 | 4405875 | 50,465 | 45,418 | 1,716 |  | 934 |
| 2013 | 64,105,654 | 698,512 | 3.60 | 25,146 | 4260702 | 46,426 | 41,783 | 1,671 |  | 974 |
| 2014 | 64,596,752 | 695,233 | 3.80 | 26,419 | 3957426 | 42,592 | 38,333 | 1,619 |  | 967 |
| 2015 | 64,596,752 | 697,852 | 4.00 | 27,914 | 3290497 | 35,548 | 31,993 | 1,422 |  | 886 |
| Till Apr 2016* | 64,596,752 | 697,852 | 4.00 | 27,914 | 2871604 | 31,023* | 27,920* | 409* |  | 244* |
|  |  |  |  |  |  | 909,540 | 818,586 | **23,425** | **18,470** | **9,118** |

**S1 Table.** The total number of expectant women with gestational diabetes mellitus (GDM) during the study period was calculated as 23,425. Based on the assumption that 80/100 expectant mothers were diagnosed with GDM for the first time, a total of 18,740 women had GDM of which 49% (n=9,118) were captured in this study.
