# Supplementary material for: Increased risk of ischemic heart disease, hypertension, and type 2 diabetes in women with previous gestational diabetes mellitus, a target group in general practice for preventive interventions: A population-based cohort study
Source: PLoS Med. 2018 Jan 16;15(1):e1002488. doi: 10.1371/journal.pmed.1002488 (PMC5770032; doi:10.1371/journal.pmed.1002488)
Supplement: S1 Text — (DOCX) [file pmed.1002488.s004.DOCX]

**Protocol for examining the association between cardiometabolic outcomes in women diagnosed with gestational diabetes mellitus in a large primary care (THIN) UK dataset.**

**Background**

All women with diabetes in pregnancy require active treatment to achieve optimum serum glucose levels and improve maternal and fetal outcomes.[1-3] Most women with diabetes in pregnancy (87.5%) are diagnosed with gestational diabetes mellitus (GDM),[4] that has rapidly increased over the past few years largely due to the obesity epidemic.[1] The World Health Organisation (2010) diagnostic criteria for GDM is based on a fasting glucose >5.1mmol/L, and following a 75g oral glucose challenge, a one-hour >10mmol/L or two-hour >8.5mmol/L.[5] Although inconsistencies exist across countries for screening for GDM[1] and in diagnostic cut-off points for glycated haemoglobin (HbA_1c_),[6] the prevalence reported for Europe is 2-6%,[1] 7% for North America[7] and 10-20% in high risk populations.[8] Universal screening is recommended as early identification and treatment of women with prediabetes and type 2 diabetes in early pregnancy and GDM during the second trimester reduces pregnancy and perinatal complications,[1-3,9] increases the rate of vaginal deliveries[10] and improves infant birth weights.[10,11]

GDM is a well-established predictor for progression to type 2 diabetes,[12,13] and carries up to a 70% lifetime risk.[13] Women with GDM are more likely to have markers for insulin resistance and beta cell dysfunction[14-17] particularly if they are overweight,[18] and exhibit more cardiovascular risk factors in the post-partum period compared with women who remain normoglycemic during pregnancy.[15] Despite this, there is a paucity of reports on the association between women who are diagnosed with GDM and the development of cardiovascular disease. Additional population-based studies utilizing large routinely collected datasets would add a valuable information on the risk of long term cardiometabolic outcomes for women diagnosed with GDM and in the longer-term screening and clinical management of women diagnosed with GDM.

**Clinical Importance**

Despite the recommendation for annual screening for type 2 diabetes in women diagnosed with GDM[19] and evidence that lifestyle changes can improve outcomes[20] there is a paucity of reports on post-partum screening and low rates have been reported.[21,22] Screening for metabolic syndrome, type 2 diabetes and associated morbidities in primary care can be done with a simple and relatively inexpensive blood test for HbA1c, lipids, thyroxine (T_4_) along with assessment of blood pressure, tobacco use and menstrual irregularities. In addition, screening for cardiometabolic risk factors would ensure women knew of their increased risk and presents an opportunity to educate and support women to make the required lifestyle changes to improve outcomes,[12,16] although there is currently a lack of evidence on improving long term metabolic outcomes.[23]

**Methods**

A retrospective cohort study design will be used to make comparisons between women diagnosed with GDM and those without a documented diagnosis utilizing the The Health Improvement Network (THIN) dataset. All women who have given birth between January 1990 and May 2016 will be included in the analyses. All general practices in the database that installed and fully used the computerised electronic central data recording system for at least one year, and have an accepted mortality recording (AMR) for at least 12 months, will be included in the analyses.

**Study population**

All women who have given birth during this period who were registered with an eligible practice for at least nine months prior to giving birth and remain for at least the 6-week follow-up post-partum consult will be included in the analyses. All eligible women will be included in the initial univariate analyses to report trends in GDM, type 1 and type 2 diabetes and documented risk factors. Women diagnosed with GDM and type 2 diabetes will be separately compared with women who did not have a documented diagnosis of diabetes during pregnancy.

**Outcomes**

Cardiovascular outcome (ischemic heart disease (IHD)) and cerebrovascular disease (stroke or transient ischemia attack).

Hypertension

Type 2 diabetes.

**Data analyses**

Women with GDM will be matched with control women in the cohort and comparisons made for baseline characteristics. Incidence rate ratios (IRR) and 95% confidence intervals (CI) will be calculated All analysis will be conducted using STATA 14·0 [24].

**References**

1. Buckley BS, Harreiter J, Damm P, Corcoy R, Chico A, Simmons D, et al. Gestational diabetes mellitus in Europe: prevalence, current screening practice and barriers to screening. A review. Diabet Med. 2012;29(7):844-54. doi: 10.1111/j.1464-5491.2011.03541.x. PubMed PMID: 22150506.

2. Simmons D. Diabetes and obesity in pregnancy. Best practice & research Clinical obstetrics & gynaecology. 2011;25(1):25-36. doi: 10.1016/j.bpobgyn.2010.10.006. PubMed PMID: 21247811.

3. Hughes RC, Moore MP, Gullam JE, Mohamed K, Rowan J. An early pregnancy HbA1c >/=5.9% (41 mmol/mol) is optimal for detecting diabetes and identifies women at increased risk of adverse pregnancy outcomes. Diabetes Care. 2014;37(11):2953-9. doi: 10.2337/dc14-1312. PubMed PMID: 25190675.

4. NICE. Diabetes in pregnancy: management from preconception to the postnatal period United Kingdom2015 [cited 2017 4 August]. Available from: <https://www.nice.org.uk/guidance/ng3/resources/diabetes-in-pregnancy-management-from-preconception-to-the-postnatal-period-51038446021>.

5. World Health Organization. Diagnostic Criteria and Classification of Hyperglycaemia First Detected in Pregnancy. Geneva (Switzerland): World Health Organization, 2013.

6. Agarwal MM, Dhatt GS, Othman Y. Gestational diabetes: differences between the current international diagnostic criteria and implications of switching to IADPSG. J Diabetes Complications. 2015;29(4):544-9. doi: 10.1016/j.jdiacomp.2015.03.006. PubMed PMID: 25837380.

7. American Diabetes A. Diagnosis and classification of diabetes mellitus. Diabetes Care. 2014;37 Suppl 1:S81-90. doi: 10.2337/dc14-S081. PubMed PMID: 24357215.

8. Galtier F. Definition, epidemiology, risk factors. Diabetes Metab. 2010;36(6 Pt 2):628-51. doi: 10.1016/j.diabet.2010.11.014. PubMed PMID: 21163426.

9. Simmons D, Rowan J, Reid R, Campbell N, National GDMWP. Screening, diagnosis and services for women with gestational diabetes mellitus (GDM) in New Zealand: a technical report from the National GDM Technical Working Party. NZMJ. 2008;121(1270):74-86. PubMed PMID: 18364758.

10. Griffin ME, Coffey M, Johnson H, Scanlon P, Foley M, Stronge J, et al. Universal vs. risk factor-based screening for gestational diabetes mellitus: detection rates, gestation at diagnosis and outcome. Diabet Med. 2000;17(1):26-32. PubMed PMID: 10691156.

11. Rowan JA, Budden A, Ivanova V, Hughes RC, Sadler LC. Women with an HbA of 41-49 mmol/mol (5.9-6.6%): a higher risk subgroup that may benefit from early pregnancy intervention. Diabet Med. 2015. doi: 10.1111/dme.12812. PubMed PMID: 26031320.

12. Kramer CK, Swaminathan B, Hanley AJ, Connelly PW, Sermer M, Zinman B, et al. Each degree of glucose intolerance in pregnancy predicts distinct trajectories of beta-cell function, insulin sensitivity, and glycemia in the first 3 years postpartum. Diabetes Care. 2014;37(12):3262-9. doi: 10.2337/dc14-1529. PubMed PMID: 25231898.

13. Kim C, Newton KM, Knopp RH. Gestational diabetes and the incidence of type 2 diabetes: a systematic review. Diabetes Care. 2002;25(10):1862-8. PubMed PMID: 12351492.

14. Li W, Zhang S, Liu H, Wang L, Zhang C, Leng J, et al. Different associations of diabetes with beta-cell dysfunction and insulin resistance among obese and nonobese Chinese women with prior gestational diabetes mellitus. Diabetes Care. 2014;37(9):2533-9. doi: 10.2337/dc14-0573. PubMed PMID: 24914241.

15. Sokup A, Ruszkowska-Ciastek B, Walentowicz-Sadlecka M, Grabiec M, Rosc D. Gestational diabetes mellitus worsens the profile of cardiometabolic risk markers and decrease indexes of beta-cell function independently of insulin resistance in nondiabetic women with a parental history of type 2 diabetes. Journal of diabetes research. 2014;2014:743495. doi: 10.1155/2014/743495. PubMed PMID: 25097861; PubMed Central PMCID: PMCPMC4109116.

16. Kim C. Maternal outcomes and follow-up after gestational diabetes mellitus. Diabet Med. 2014;31(3):292-301. doi: 10.1111/dme.12382. PubMed PMID: 24341443; PubMed Central PMCID: PMCPMC3944879.

17. Kusunoki Y, Katsuno T, Nakae R, Watanabe K, Ochi F, Tokuda M, et al. Insulin resistance and beta-cell function influence postprandial blood glucose levels in Japanese patients with gestational diabetes mellitus. Gynecol Endocrinol. 2015;31(12):929-33. doi: 10.3109/09513590.2015.1075498. PubMed PMID: 26288254.

18. Lekva T, Bollerslev J, Godang K, Roland MC, Friis CM, Voldner N, et al. beta-cell dysfunction in women with previous gestational diabetes is associated with visceral adipose tissue distribution. Eur J Endocrinol. 2015;173(1):63-70. doi: 10.1530/EJE-15-0153. PubMed PMID: 25877991.

19. Kalra B, Gupta Y, Kalra S. Gestational diabetes mellitus (GDM) follow up: as simple as ABCDE. Diabetes Res Clin Pract. 2015;107(2):e5-6. doi: 10.1016/j.diabres.2014.12.006. PubMed PMID: 25624178.

20. Smith BJ, Cinnadaio N, Cheung NW, Bauman A, Tapsell LC, van der Ploeg HP. Investigation of a lifestyle change strategy for high-risk women with a history of gestational diabetes. Diabetes Res Clin Pract. 2014;106(3):e60-3. doi: 10.1016/j.diabres.2014.09.035. PubMed PMID: 25451910.

21. Chang Y, Chen X, Cui H, Zhang Z, Cheng L. Follow-up of postpartum women with gestational diabetes mellitus (GDM). Diabetes Res Clin Pract. 2014;106(2):236-40. doi: 10.1016/j.diabres.2014.08.020. PubMed PMID: 25271112.

22. Paez KA, Eggleston EM, Griffey SJ, Farrar B, Smith J, Thompson J, et al. Understanding why some women with a history of gestational diabetes do not get tested for diabetes. Womens Health Issues. 2014;24(4):e373-9. doi: 10.1016/j.whi.2014.04.008. PubMed PMID: 24981396.

23. Hartling L, Dryden DM, Guthrie A, Muise M, Vandermeer B, Aktary WM, et al. Screening and diagnosing gestational diabetes mellitus. Evidence report/technology assessment. 2012;(210):1-327. PubMed PMID: 24423035.

24. StataCorp. Stata Statistical Software: Release 14 College Station, TX: StataCorp LP2015 [cited 2017 7 May]. Available from: <http://www.stata.com/>.
